# Application of lacrimal gland ultrasonography in the evaluation of chronic ocular graft-versus-host-disease

**DOI:** 10.3389/fimmu.2025.1490390

**Published:** 2025-02-05

**Authors:** Mingxia Zhong, Siyuan Liu, Jinghan Luo, Qin Zhang, Zhou Yang, Shanshan Zhang

**Affiliations:** ^1^ Peking University People’s Hospital, Beijing, China; ^2^ Department of Ophthalmology, Peking University People's Hospital, Beijing, China; ^3^ School of Public Health, Peking University, Beijing, China; ^4^ Department of Ultrasound, Peking University People's Hospital, Beijing, China

**Keywords:** ocular graft-versus-host-disease, B-mode ultrasonography, lacrimal gland, dry eye disease, ocular surface status

## Abstract

**Objective:**

To investigate the effectiveness of lacrimal gland ultrasonography in the assessment of chronic ocular graft-versus-host-disease (oGVHD) after allogeneic hematopoietic stem cell transplantation (allo-HSCT) and to establish the correlation between the ocular surface and ultrasonographic results.

**Method:**

The cross-sectional study included 57 participants aged 18 and older, who were at least 100 days after allo-HSCT. The study was conducted at the oGVHD clinic of Peking University People’s Hospital between March to June 2023. Patients were categorized into groups according to the International Chronic oGVHD (ICCGVHD) consensus group diagnostic criteria or the 2005 National Institutes of Health (NIH) classification criteria for Chronic GVHD. Demographics and transplantation-related information were collected for all participants, including age, gender, donor-recipient HLA matching, donor-recipient ABO matching, donor-recipient gender combination and duration after allo-HSCT. The disease activity of oGVHD and the severity of ocular surface involvement were assessed using various parameters such as Ocular Surface Disease Index (OSDI), Schirmer test, tear film break-up time (BUT), tear meniscus height, corneal/conjunctival staining and meibomian gland dropout. Lacrimal gland structures were assessed by B-mode and Doppler ultrasonography to measure parameters such as the long diameter, thick diameter, homogeneity and parenchymal vascularization. Statistical analyses were performed to determine differences in ocular surface conditions and lacrimal gland ultrasonographic parameters between groups as well as to determine the correlation between ocular surface condition and lacrimal gland ultrasonographic findings.

**Result:**

(1) Patients with definite and probable oGVHD exhibited a significantly longer duration after allo-HSCT compared to non-oGVHD patients (H=11.264, p<0.01), The median durations were 247(164,894) days and 525(310,928) days, respectively, compared to 204(169,323.25) days for non-oGVHD patients. (2) Compared to non-oGVHD patients, both definite oGVHD patients and probable oGVHD patients showed lower average of Schirmer test (H=31.188, p<0.01), TBUT (H=11.853, p<0.01), tear meniscus height (H=13.630, p<0.01) and higher average of OSDI (F=27.992, p<0.01), corneal staining scores (χ²=23.66, p<0.05) and temporal conjunctival staining scores (χ²=14.84, p<0.05). (3) The B-mode and Doppler ultrasonography parameters in lacrimal glands including long diameter, thick diameter, homogeneity and parenchymal vascularization did not exhibit significant differences between the three groups. (4) The long diameter in lacrimal ultrasonography had significantly positive correlations with tear meniscus height (r=0.297, p<0.05) and significantly negative correlations with temporal conjunctival staining scores (r=-0.313, p<0.05) and staining total scores (r=-0.285, p<0.05). The thick diameter in lacrimal ultrasonography demonstrated significantly positive correlations with tear meniscus height (r=0.404, p<0.01), and significantly negative correlations with OSDI (r=-0.273, p<0.05), corneal staining scores (r=-0.264, p<0.05), nasal conjunctival staining scores (r=-0.271, p<0.05) and staining total scores (r=-0.312, p<0.05). Homogeneity and parenchymal vascularization were not found to be significantly correlated with ocular surface status.

**Conclusion:**

The ocular surface condition in oGVHD patients is worse than that observed in non-GVHD patients. The main manifestations include keratoconjunctival injury and a reduction in tear secretion and tear film stability. These effects appear to be a common result of chemoradiotherapy-induced inflammation and rejection-associated responses. There were no significant differences in the morphology of lacrimal glands as revealed by ultrasonography. This suggests that ocular rejection may not be the primary cause of lacrimal gland changes in oGVHD patients. While ultrasonography can provide insight into tear secretion, its efficacy in diagnosing oGVHD appears limited.

## 1 Introduction

For various hematologic malignancies and severe immunodeficiencies, allogeneic hematopoietic stem cell transplantation (allo-HSCT) is a curative treatment modality (1). Annually, approximately 30,000 allo-HSCT procedures are conducted worldwide, with a consistent upward trend in transplant numbers (1, 2). Graft-versus-host disease (GVHD), which is the major complication of allo-HSCT, can affect many tissues including the skin, liver, gut and eye, increasing the risk of morbidity and mortality in the post-treatment period (3, 4). GVHD is a multi-organ disease derived from immune dysregulation and tissue inflammation with single or multisystem involvement, resulting in tissue fibrosis and organ dysfunction (5). GVHD can occur in 10~80% of transplant recipients with ocular involvement observed in 40~60% of cases (6, 7), which indicates that oGVHD is more common in patients after transplantation and has certain indicative significance for rejection after transplantation. GVHD involving the eyes is generally a chronic disease. Chronic graft-versus-host disease-related dry eye (cGVHD-DE) is the most common manifestation of oGVHD (8), which is manifested as dry eye, foreign body sensation, severe light sensitivity, chronic conjunctivitis, periorbital hyperpigmentation, itching and eye tingling after allo-HSCT, resulting in obvious eye discomfort, decreased vision, and even blindness (9–11).

At present, the widely recognized diagnostic criteria for oGVHD include The National Institutes of Health Consensus Conference (NIH CC) 2014 criteria (9) and The International Chronic oGVHD (ICCGVHD) consensus group diagnostic criteria (12). A comparative study of the NIH 2014 criteria and ICCGVHD criteria found that the two have a moderate agreement, but the ICCGVHD criteria were noted to be better at differentiating oGVHD patients from non‐oGVHD dry eye disease (DED), due to its more stringent criteria which also considers the status of systemic GVHD (13). The diagnosis of chronic oGVHD(coGVHD) is mainly based on the presence of ocular manifestations such as dry eye and ocular surface damage. However, the feature of dry eye in oGVHD overlaps those of dry eye disease though they have distinct etiologies, presentation, pathophysiology, clinical manifestations, and treatments (14). Tears are secreted by the lacrimal gland, and a reduction in tear production can precede the onset of dry eye. Consequently, changes in the morphology of the lacrimal gland may occur before the manifestation of dry eye. As the disease progresses to advanced stages, suffering a significant decline in the quality of life and limited effective treatment options potentially impose a substantial financial burden on their families. Therefore, it is of great significance and importance to find more reasonable and integrated methods for early and objective diagnosis of oGVHD (13).

Another condition characterized by dry eye as its primary clinical manifestation is Sjogren’s disease (SjD), a chronic autoimmune connective tissue disease presenting a triad of symptoms including sicca symptoms, fatigue, and pain (15). Sjogren’s disease is histopathologically characterized by lymphocytic infiltration of exocrine glands including lacrimal leading to dry eyes(keratoconjunctivitis sicca) (16, 17). Studies using mouse models of chronic GVHD have revealed inflammatory changes in lacrimal glands, featuring ductal epithelia inflammatory cell infiltration, including eosinophils, macrophages, CD8^+^T cells together with some CD4^+^T cells and finally leading to fibrosis around the lacrimal gland ducts (18, 19). A recent research observed a reduced proportion of epithelial cell populations and different gene expressions in GVHD lacrimal glands compared with non-GVHD, strengthening the relationship between the lacrimal gland and the development of oGVHD (20). Interestingly, the histological characteristics of lacrimal gland involvement in Sjogren’s disease and oGVHD appear similar. Ultrasonography (US) is widely used in the diagnosis of numerous diseases due to its non-invasive nature, ease of use, widespread availability, and real-time assessment capabilities. Salivary gland ultrasonography is commonly used to evaluate major salivary gland involvement in Sjogren’s disease (21). Although the relationship between lacrimal gland ultrasound(LGUS) characteristics and lacrimal gland (LG) histology remains unclear (22), a few studies have examined LGUS abnormalities, including fibrous gland appearance, heterogeneous texture, enlarged masses of cystic structures, and/or reticulated appearance (23–26). While studies have explored the application of ultrasound in evaluating conditions such as Sjogren’s disease, there is a notable absence of research on its utilization in the assessment of oGVHD. Furthermore, the correlation between lacrimal gland morphology and ocular surface clinical indicators in oGVHD remains unexplored. Compared with other evaluation tools mentioned in NIH CC 2014 or ICCGVHD criteria which focus on dry eye and ocular surface damage, lacrimal gland ultrasound may reduce the overlap with the diagnosis of dry eye disease and find some earlier changes in the lacrimal gland. Therefore, it is significant and possibly feasible to explore major lacrimal gland involvement by lacrimal gland ultrasonography in oGVHD. This study aims to investigate the effectiveness of lacrimal gland ultrasonography in assessing coGVHD following allogeneic hematopoietic stem cell transplantation (allo-HSCT) and determine the association between clinical ocular surface activity and ultrasonographic findings.

## 2 Patients and methods

The procedures conducted for the study are consistent with the Helsinki Declaration and approved by the Ethics Committee of Peking University People’s Hospital. All subjects have been informed of the aim of the study, the principles of related examination methods and possible adverse consequences. Each individual signed an informed consent under total comprehension.

### 2.1 Research objects

The sample size was based on the ability to detect a correlation with an absolute value ≥0.40. at a two-sided α level of 0.05 and with 80% power. A minimum of 46 patients was needed to meet our analysis.

57 patients after allo-HSCT who were treated at the eye rejection clinic of Peking University People’s Hospital from March to June 2023 were enrolled. All the patients were Chinese Han nationality.

#### 2.1.1 Inclusion criteria

1. older than 18 years old and
2. more than 100 days after allo-HSCT.

#### 2.1.2 Exclusion criteria

1. previously suffered from ocular surface diseases such as conjunctivitis, keratitis, dry eye and dacryoadenitis.
2. previously suffered from glaucoma, uveitis and retinopathy.
3. Previously taken drugs that could influence tear secretion or ocular surface injury based on Dry Eye Syndrome Preferred Practice Pattern 2024 from American Academy of Ophthalmology (AAO) (27).
4. diagnosed autoimmune diseases such as Sjogren’s disease (SjD) (criteria from ACR/EULAR 2016), systemic lupus erythematosus (SLE) (criteria from ACR/EULAR 2019) and rheumatoid arthritis (RA) (criteria from ACR/EULAR 2010).
5. history of eye surgery or trauma.
6. psychopaths.

The two diagnostic criteria for oGVHD are as follows:

1. The International Chronic oGVHD (ICCGVHD) consensus group diagnostic criteria (19): The diagnostic criteria are based on scores derived from the Ocular Surface Disease Index (OSDI), Schirmer’s test (SIT) without anesthesia, corneal lissamine green staining, conjunctival injection, and presence of systemic GVHD. The diagnostic categories included no oGVHD, probable oGVHD, and definite oGVHD.
2. The National Institutes of Health Consensus Conference (NIH CC) 2014 criteria (9): The diagnostic criteria are based on Schirmer’s test and slit−lamp examination.

According to the above two classification criteria, patients were categorized respectively and subsequent group differences were subject to analysis. NIH’s classification of oGVHD predominantly relies on the reduction of tear volume as an indicator of disease severity. However, this classification’s limitation lies in its exclusion of factors such as inflammatory activity or the extent of ocular surface disease, including corneal and conjunctival involvement, as well as the patient’s subjective experience of dry eye. Consequently, it lacks substantive guidance for treatment decisions and a comprehensive assessment of disease severity. Considering that the two criteria have a moderate agreement and the ICCGVHD criteria is better at differentiating oGVHD patients from non‐oGVHD DED due to its more stringent criteria, our primary focus in this analysis rests on the ICCGVHD criteria, while the data analysis based on the NIH CC 2014 criteria has been detailed in the **Supplementary Data**.

### 2.2 Ocular surface and tear assessment

In addition to the routine ophthalmic examination of sight and intra-ocular tension, ocular Surface Disease Index (OSDI), Schirmer test, tear film break-up time (TBUT), tear meniscus height, corneal/conjunctival staining and meibomian gland dropout were applied to evaluate the ocular surface and tear film function of the patients.

#### 2.2.1 OSDI

The OSDI is a 12-item questionnaire assessing the symptoms of ocular irritation associated with dry eye and their visual function. The questionnaire covers ocular symptoms, vision-related functions and environmental trigger factors. Patients score the severity of each symptom on a Likert scale from 0 (never) to 4 (always). The total OSDI score is calculated as follows: [(total score of all answered questions × 25)]/[(total number of questions answered)].

#### 2.2.2 Schirmer test

The Schirmer I test was applied to detect the secretory function of the lacrimal gland. Two 5mm × 35mm filter papers were placed at the junction of the inner 1/3 and the middle 1/3 of the palpebral fissure without anesthetics. The wet length of the filter paper was checked after 5 minutes of closed-eye clamping.

#### 2.2.3 TBUT

The patients were evenly stained with sodium ophthalmic test paper and were asked to blink once and then keep her/his eyes open. The time in seconds between the patient’s last blink and the first dry spot on the corneal surface was recorded under the cobalt blue light of the slit lamp. It was obtained by repeating 3 times and the mean value was measured.

#### 2.2.4 Corneal/conjunctival staining

The corneal and conjunctival lissamine green staining test paper was used to uniformly stain the ocular surface of the patient and then the corneal and conjunctival staining was observed by slit lamp microscope(SLM). The corneal staining scores, nasal conjunctival staining scores, temporal conjunctival staining scores and the total staining scores were evaluated and recorded respectively according to SICCA Ocular Staining Score (OSS) by only one experienced observer (28).

#### 2.2.5 Meibomian dropout

Non-contact infra-red meibography was performed on patients using the portable non-contact meibograph (PNCM). Meibography images were classified by only one experience. Each image was classified applying a four grade scale (29) (loss rate: degree 0 = no partial glands; 1 = <25% partial glands; 3 = 25–75% partial glands; 3 = >75% partial glands) applying ImageJ 1.42q (Wayne Rasband, National Institute of Health, USA; http://rsbweb.nih.gov/ij/).

### 2.3 Lacrimal gland ultrasonography

**Figure 1:**
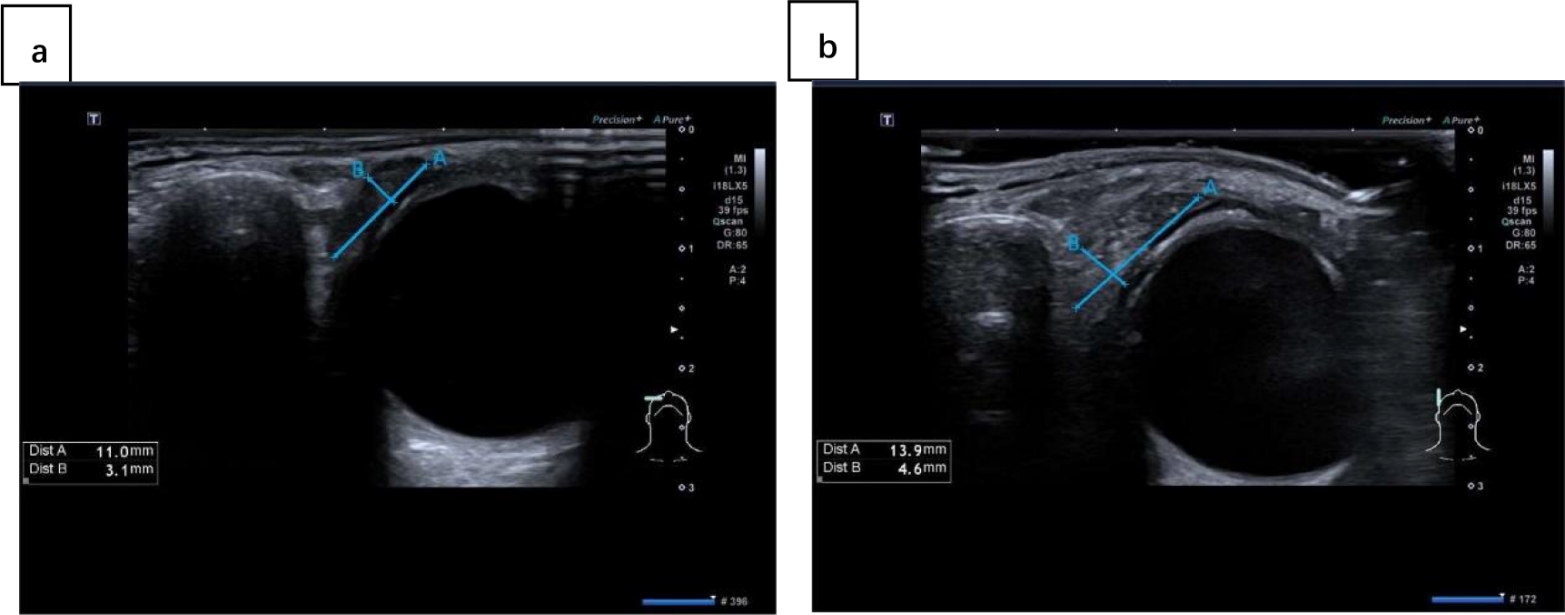
The ultrasound images of lacrimal gland in different planes: **(A)** transverse plane; **(B)** vertical plane. Line A represents the long diameter and line B represents the thick diameter.

US examinations of lacrimal and salivary glands were performed by a single radiologist. The machine used for the experiment is an ultrasound scanner (Aplio i900; Canon Medical Systems Corporation, Otawara, Tochigi, Japan) equipped with a linear array transducer (18 MHz). The radiologist was blinded to the clinical data of the subjects. The US of the lacrimal gland was performed while the subjects were supine, the head turned to the contralateral side, and the neck hyperextended. The participants were advised to breathe normally with their eyes closed so that the lacrimal gland could be located between the end of the eyelid and the eyebrow. Measurements were taken on the screen where the lacrimal gland appeared the largest. Bilateral lacrimal glands were assessed consecutively. We measured both vertical and transverse planes. Refer to some previous studies on lacrimal gland ultrasound (24, 26, 30), we evaluated the following US parameters:(1) size (long & thick diameter) (**Figure 1**); (2) homogeneity (homogenous/heterogeneous); (3) parenchymal vascularization (normal/increased).

### 2.4 Statistical analysis

For all data based on ocular surface and tear assessments, we used data from the right eye for analysis. Analyses were performed using the software SPSS statistical package (v. 24.0 for Windows, SPSS). Data were analyzed using descriptive statistics. Normality of the distribution was performed using the Kolmogorov-Smirnov test for numerical data. Numerical data conforming to normal distribution were expressed as mean ± standard deviation and T test or one-way analysis of variance was used for comparison between groups. Numerical data conforming to biased distribution were expressed as median with interquartile deviation and Kruskal-Wallis H-test was used for comparison between groups. For categorical data, χ² test or Fisher’s exact test was used for comparison between groups. Pearson correlation analysis was performed to assess the correlation of US parameters and ocular surface status when data was numerical and conforming to normal distribution. For categorical data or numerical data conforming to biased distribution, spearman correlation analysis was applied to assess the correlation.

## 3 Results

Fifty-seven patients (26 females, 31 males; age range from 18 to 65 years old; averaged 40.13 ± 11.074 years old) were included.

### 3.1 Analysis of differences between groups classified by ICCGVHD criteria

All patients were assigned to three different groups (definite oGVHD, probable oGVHD and non-oGVHD). The Demographics and transplantation characteristics of the patients are given in **Table 1**. Duration after allo-HSCT was remarkably longer in definite oGVHD group and probable oGVHD group than non-oGVHD group (median with interquartile deviation: 247 (164,894) and 525(310,928) vs 204(169,323.25), H=11.264, p<0.01). There was no significant difference in age, gender, donor-recipient gender combination, HLA matching, and ABO matching between the three groups.

**Table 1:** Demographics and transplantation characteristics of patients after allo-HSCT classified by ICCGVHD criteria.

Variables	Definite oGVHD (n=12)	Probable oGVHD (n=28)	Non-oGVHD (n=17)	H/F/χ²	P-value
**Age (years)**	38.6 ± 17.126	40.6 ± 8.559	40.17 ± 12.014	F=0.208	0.813
**Gender**				χ²=0.193	0.908
** Female**	6 (50.0%)	12 (42.9%)	8 (47.1%)		
** Male**	6 (50.0%)	16 (57.1%)	9 (52.9%)		
**Duration after allo-HSCT**	247 (164,894)	525 (310,928)	204 (169,323.25)	H=11.264	0.004^**^
**Donor-recipient HLA matching, n (%)**				χ²=0.902	0.637
** Related HLA-identical donor**	4 (33.3%)	12 (42.9%)	5 (29.4%)		
** Haplp-identical family donor**	8 (66.7%)	16 (57.1%)	12 (70.6%)		
**Donor-recipient ABO matching, n (%)**				χ²=0.420	0.999
** ABO-compatible**	6 (50%)	13 (46.4%)	8 (47.1%)		
** Major ABO-incompatible**	4 (33.3%)	9 (32.1%)	6 (35.3%)		
** Minor ABO-incompatible**	1 (8.3%)	3 (10.7%)	1 (5.9%)		
** Major&minor ABO-incompatible**	1 (8.3%)	3 (10.7%)	2 (11.8%)		
**Donor-recipient gender combination, n (%)**				χ²=6.420	0.378
** Male to male**	1 (8.3%)	3 (11.5%)	6 (37.5)		
** Female to female**	2 (16.7%)	3 (11.5%)	2 (12.5%)		
** Male to female**	4 (33.3%)	9 (34.6%)	5 (31.3%)		
** Female to male**	5 (41.7%)	11 (42.3%)	3 ( (18.8%)		
** Missing**		2 (7.7%)	1 (6.3%)		

H, the result of Kruskal-Wallis H-test; F, the result of one-way analysis of variance; χ², the result of χ² test. The same for the following tables. The bold means p value < 0.05. The symbol "**" means p value <0.01.

Compared to non-oGVHD patients, both definite oGVHD patients and probable oGVHD patients showed a lower average of Schirmer test (H=31.188, p<0.01), TBUT (H=11.853, p<0.01), tear meniscus height (H=13.630, p<0.01) and a higher average of OSDI (F=27.992, p<0.01), corneal staining scores (χ²=23.66, p<0.05) and temporal conjunctival staining scores (χ²=14.84, p<0.05) (**Table 2**), which determined a severe condition of the ocular surface. However, the index of meibomian gland dropout and its grade did not show inter-group differences (p>0.05).

**Table 2:** Ophthalmic parameters of patients after allo-HSCT classified by ICCGVHD criteria.

Variables	Definite oGVHD (n=12)	Probable oGVHD (n=28)	Non-oGVHD (n=17)	H/F/χ²	P-value
**Sight**	0.80(0.35,1.10)	0.60(0.50,1.00)	1.00(0.60,1.00)	H=4.083	0.130
**intra-ocular tension**	16.20(11.5,19.00)	16.00(14.00,19.00)	13.75(12.00,18.00)	H=1.647	0.439
**OSDI**	22.78 ± 11.79	48.34 ± 22.01	12.45 ± 9.76	F=27.992	<0.001^**^
**Schirmer test**	8.00(4.00,10.00)	2.00(1.00,5.00)	14.50(8.75,18.50)	H=31.188	<0.001^**^
**TBUT**	4.00(2.75,8.25)	2.00(1.00,3.00)	4.00(3.00,5.50)	H=11.853	0.003^**^
**Tear meniscus height**	0.16(0.12,0.23)	0.14(0.11,0.18)	0.20(0.15,0.25)	H=13.630	0.001^**^
**Corneal staining scores(≤6)**				χ²=23.66	0.023^*^
** 0**	7(58.3%)	7(25%)	15(88.2%)		
** 1~3**	3(25.0%)	8(28.6%)	1(5.9%)		
** ≥4**	2(16.7%)	13(46.4%)	1(5.9%)		
**Nasal conjunctival staining scores(≤3)**				χ²=9.151	0.165
** 0**	4(33.3%)	3(10.7%)	8(47.1%)		
** 1~2**	4(33.3%)	8(28.6%)	3(17.6%)		
** 3**	4(33.3%)	17(60.7%)	6(35.3%)		
**Temporal conjunctival staining scores(≤3)**				χ²=14.84	0.022^*^
** 0**	5(41.7%)	6(21.4%)	12(70.6%)		
** 1~2**	4(33.3%)	7(25.0%)	4(23.5%)		
** 3**	3(25.0%)	15(53.6%)	1(5.9%)		
**staining total scores(≤12)**				χ²=31.944	0.078
** 0**	2(16.7%)	1(3.6%)	6(35.3%)		
** 1~4**	6(50.0%)	5(17.9%)	9(52.9%)		
** 5~9**	3(25.0%)	15(53.6%)	2(5.9%)		
** ≥10**	1(8.3%)	7(25.0%)	0(0%)		
**Meibomian**	33.30(25.85,38.95)	41.80(31.10,83.70)	39.85(20.63,64.05)	H=0.752	0.687
**Grade of meibomian gland dropout(≤4)**				χ²=6.752	0.564
** 0**	0(0%)	0(0%)	1(5.9%)		
** 1~2**	10(83.3%)	17(60.7%)	12(70.6%)		
** 3~4**	2(16.7%)	9(32.1%)	4(23.5%)		
** Missing**		2(7.1%)			

The bold means p value < 0.05. The symbol "*" means p value <0.05 and the symbol "**" means p value <0.01.

The B-mode and Doppler ultrasonography parameters in lacrimal glands including long diameter, thick diameter, homogeneity and parenchymal vascularization did not differ between the three groups (**Table 3**).

**Table 3:** B-mode and Doppler ultrasonography evaluations of patients after allo-HSCT classified by ICCGVHD criteria.

Variables	Definite oGVHD (n=12)	Probable oGVHD (n=28)	Non-oGVHD (n=17)	H/F/χ²	P-value
**Long diameter**	8.620 ± 1.278	8.687 ± 1.166	10.117 ± 2.459	F=2.807	0.069
**Thick diameter**	3.400 ± 0.943	3.300 ± 0.674	3.983 ± 1.202	F=2.807	0.128
**Homogeneity**				χ²=2.857	0.240
** Homogenous**	1 (8.3%)	2 (7.1%)	4 (23.5%)		
** Heterogeneous**	11 (91.7%)	26 (92.9%)	13 (76.5%)		
**Parenchymal vascularization**				χ²=1.849	0.397
** Normal**	11 (91.7%)	25 (89.3%)	13 (76.5%)		
** Increased/decreased**	1 (8.3%)	3 (10.7%)	4 (23.5%)		

The bold means p value < 0.05.

### 3.2 Correlation analysis of lacrimal ultrasonography and dry eye tests

The long diameter in lacrimal ultrasonography had significantly positive correlations with tear meniscus height (r=0.297, p<0.05) and significantly negative correlations with temporal conjunctival staining scores (r=-0.313, p<0.05) and staining total scores (r=-0.285, p<0.05). The thick diameter in lacrimal ultrasonography had significantly positive correlations with tear meniscus height (r=0.404, p<0.01), and significantly negative correlations with OSDI (r=-0.273, p<0.05), corneal staining scores (r=-0.264, p<0.05), nasal conjunctival staining scores (r=-0.271, p<0.05) and staining total scores (r=-0.312, p<0.05). Homogeneity and parenchymal vascularization were not found to be significantly correlated with ocular surface status. (**Table 4**).

**Table 4:** Correlation analysis of quantitative parameters of B-mode and Doppler ultrasonography in lacrimal glands and dry eye tests.

Parameters	Long diameter		Thick diameter		Homogeneity		Parenchymal vascularization	
	r	p	r	p	r	p	r	p
**Sight**	0.061	0.699	0.224	0.154	-0.078	0.618	0.050	0.752
**intra-ocular tension**	0.032	0.824	0.141	0.319	0.099	0.479	-0.133	0.343
**OSDI**	-0.245	0.069	-0.273	0.042^*^	0.014	0.917	-0.103	0.452
**Schirmer test**	0.086	0.534	0.184	0.178	-0.065	0.632	0.087	0.523
**TBUT**	0.067	0.689	-0.021	0.901	-0.249	0.126	-0.042	0.799
**Tear meniscus height**	0.297	0.026^*^	0.404	0.002^**^	-.183	0.878	-0.055	0.685
**Corneal staining scores**	-0.172	0.205	-0.264	0.049^*^	-0.014	0.918	0.046	0.732
**Nasal conjunctival staining scores**	-0.241	0.074	-0.271	0.044^*^	-0.105	0.438	0.030	0.826
**Temporal conjunctival staining scores**	-0.313	0.019^*^	-0.204	0.132	-0.010	0.939	0.000	1.000
**staining total scores**	-0.285	0.033^*^	-0.312	0.019^*^	-0.083	0.537	0.028	0.837
**Meibomian gland dropout**	0.156	0.251	0.096	0.480	0.097	0.471	0.127	0.345
**Grade of meibomian gland dropout**	0.033	0.813	-0.031	0.826	0.095	0.491	0.084	0.541

The bold means p value < 0.05. The symbol "*" means p value <0.05 and the symbol "**" means p value <0.01.

## 4 Discussion

In this study, we evaluated the lacrimal gland involvement in oGVHD patients with ultrasonography. Our results suggest that lacrimal ultrasonography could be a non-invasive adjunctive tool for clinical assessment. Specifically, it proves valuable in evaluating the severity of dry eye, encompassing parameters such as tear secretion, as well as assessing the extent of corneal and conjunctival injury.

GVHD is the major complication following allo-HSCT and coGVHD emerges in 40-60% of patients. This condition is driven by complex interactions between the immune systems of the donor and the recipient, involving the recognition of host antigens by donor-derived CD4+ and CD8+ T cells (31). Dry eye disease is the hallmark of oGVHD and may be associated with inflammatory damage and fibrosis affecting the entire ocular surface system, including lacrimal and meibomian glands, cornea, conjunctiva, and eyelids (32, 33).

It has been established that the development of oGVHD is influenced by several factors linked to both donor and recipient characteristics, such as the human leukocyte antigen (HLA) mismatch or an unrelated donor, ABO mismatched and male recipients of female donors (34–36). However, in our study, aside from the duration after allo-HSCT, we did not observe significant differences in the aforementioned demographic and transplantation-related factors between oGVHD patients and non-oGVHD patients. This finding suggests that, in the context of chronic oGVHD, the duration after transplantation exerts a more pronounced impact on the deterioration of ocular surface conditions, including inflammatory damage and fibrosis while other factors mainly cause acute damage.

oGVHD most commonly involves changes to the ocular surface and can be characterized by keratoconjunctivitis sicca (or dry eye), as well as inflammatory damage to the conjunctiva, and punctate keratopathy (3, 10). It also involves inflammation of the lacrimal gland and eyelids, which is characterized by a decrease in tear secretion and tear film stability, as well as meibomian gland dropout (3, 37). In our study, parameters such as the OSDI, Schirmer test, TBUT, Tear meniscus height and corneal/conjunctival staining demonstrated significant differences between groups. This indicates that patients with oGVHD experience a reduction in tear secretion, impaired tear film stability and injury to the conjunctiva and cornea. However, the index of meibomian gland dropout and its grade did not show inter-group differences (p>0.05). This suggests that the impairment of the meibomian gland may occur in hematological patients already before HSCT, probably as the result of a multifactorial process caused by the concomitant therapies (i.e., chemo/radiotherapy) and/or the underlying disease itself with infiltration of the glands by tumor cells (37–39). As the majority of patients undergoing allo-HSCT have hematological malignancies, the occurrence of ocular toxicity induced by chemotherapeutic agents is noteworthy. Chemotherapy regimens can lead to a broad spectrum of ocular disorders such as dry eye, keratitis and lens disorders (40–42). Importantly, some of the mechanisms underlying such damage involve inflammatory reactions, similar to rejection. Thus, recognizing the inflammatory response’s role in ocular surface and lacrimal gland involvement underscores the impact of radiotherapy and chemotherapy, highlighting their significance in the context of ocular complications.

B-mode US can yield information on homogeneity, echogenicity, borders and parenchymal changes, such as intraparenchymal lymph nodes (30). Common parameters in lacrimal ultrasound are size, echogenicity, texture and parenchymal vascularization (27, 44). Although lacrimal ultrasound has been used in the assessment and even diagnosis of pSS, there is no related research applied in oGVHD. In our study, we apply B-mode and Doppler ultrasonography to evaluate the morphology, homogeneity and parenchymal vascularization. The confounding variables such as age and gender were matched between the groups. Most of the clinical indicators of the eyes were significantly different, but there was no significant difference in the results of lacrimal gland ultrasonography, which indicates that ocular rejection did not affect the morphology of the lacrimal gland under ultrasound. Here are some possible interpretations for the no difference between groups. Firstly, fibrosis and inflammation caused by stromal fibroblasts with T‐cell infiltration centers around the periductal area of the lacrimal gland are the main reasons for lacrimal gland dysfunction in oGVHD (43). However, the fibrosis is a relatively chronic process. Since studies that have examined lacrimal gland ultrasound abnormalities mentioned SjD develops over time and establishing a typical clinical picture typically takes years, the patients they enrolled turned out to have a long disease duration of years (>4 years 52.2%), which is significantly longer than the disease duration of patients enrolled in our study. Secondly, the epithelial‐mesenchymal transition of the host cells and the fibrosis progress may be triggered by chemoradiotherapy therapy before and after the HSCT (44). It suggests that the lacrimal gland changes in morphology could also be influenced by factors other than oGVHD such as chemoradiotherapy. Although there was no significant difference between GVHD and non-GVHD patients, which may suggest that ultrasound has no good effect on the diagnosis of GVHD, the morphology examined by ultrasound including long diameter and thick diameter showed a correlation with OSDI, tear meniscus height and corneal/conjunctival lissamine green staining, indicating that ultrasound can be meaningful for clinical evaluation by indicating the severity of dry eye, tear secretion and keratoconjunctival injury. However, ultrasound parameters didn’t show a correlation with TBUT, which suggests that it can’t reflect tear film stability. In addition to Doppler ultrasound, some studies have also explored the indicators of lacrimal gland under ultrasound 2-dimensional shear wave elastography (2D-SWE), a more effective imaging technique for evaluating tumor/nodule lesions and parenchymal fibrosis. It found a good correlation and diagnostic value (26). It suggests that we can use different ultrasound imaging techniques to evaluate the lacrimal gland.

Our study has several limitations. Firstly, the present study only showed results in a cross-sectional manner. Thus, the importance of LGUS on disease progression or change of LGUS was not evaluated. As a result, we cannot definitively ascertain whether lacrimal gland injury is attributable to radiotherapy, chemotherapy, or rejection. Secondly, the sample size was relatively small. Our analysis showed the homogeneity and parenchymal vascularization parameters were not significantly correlated with ocular surface and tear assessments. However, power analysis indicated that at a two-sided α level of 0.05 and with 80% power, the current analysis could only detect correlation coefficients with absolute values ≥0.36 due to the limited sample size (data not shown). Lastly, we only divided the grading of ultrasonic indicators including homogeneity and parenchymal vascularization into normal/abnormal, and did not form a detailed grading standard for accessing.

In conclusion, our study findings suggest a correlation between the morphology of lacrimal glands and clinical severity indices in oGVHD. Consequently, Doppler ultrasonography emerges as a potential auxiliary tool for early-stage clinical assessments of oGVHD, particularly in cases presenting with extra-glandular organ involvements and borderline diagnostic findings. This suggests that Doppler ultrasonography holds promise in enhancing diagnostic precision and timely intervention for individuals at the onset of oGVHD, contributing to improved patient outcomes and management strategies.

## Data Availability

The raw data supporting the conclusions of this article will be made available by the authors, without undue reservation.

## References

[1] Hill GR Betts BC Tkachev V Kean LS Blazar BR. Current concepts and advances in graft-versus-host disease immunology. Annu Rev Immunol. (2021) 39:19–49. doi: 10.1146/annurev-immunol-102119-073227 33428454 PMC8085043

[2] Nair S Vanathi M Mukhija R Tandon R Jain S Ogawa Y. Update on ocular graft-versus-host disease. Indian J Ophthalmol. (2021) 69:1038–50. doi: 10.4103/ijo.IJO_2016_20 PMC8186644 33913829

[3] Kezic JM Wiffen S Degli-Esposti M. Keeping an ‘Eye’ on ocular GVHD. Clin Exp Optom. (2022) 105:135–42. doi: 10.1080/08164622.2021.1971047 34538201

[4] Hessen M Akpek EK. Ocular graft-versus-host disease. Curr Opin Allergy Clin Immunol. (2012) 12:540. doi: 10.1097/ACI.0b013e328357b4b9 22892710

[5] Lee SJ. Classification systems for chronic graft-versus-host disease. Blood. (2017) 129:30–7. doi: 10.1182/blood-2016-07-686642 PMC5216262 27821503

[6] Aki SZ Inamoto Y Carpenter PA Storer BE Sandmaier BM Lee SJ. Confounding factors affecting the national institutes of health (NIH) chronic graft-versus-host disease organ-specific score and global severity. Bone Marrow Transplant. (2016) 51:1350–3. doi: 10.1038/bmt.2016.131 PMC5052092 27214071

[7] Justiz Vaillant AA Modi P Mohammadi O. Graft-versus-host disease. In: StatPearls. StatPearls Publishing, Treasure Island (FL (2023). 30855823

[8] Zhang CY Farooq AV Harocopos GJ Sollenberger EL Hou JH Bouchard CS. Corneal perforation in ocular graft-versus-host disease. Am J Ophthalmol Case Rep. (2021) 24:101224. doi: 10.1016/j.ajoc.2021.101224 34805617 PMC8586569

[9] Jagasia MH Greinix HT Arora M Williams KM Wolff D Cowen EW. National institutes of health consensus development project on criteria for clinical trials in chronic graft-versus-host disease: I. The 2014 diagnosis and staging working group report. Biol Blood Marrow Transplant. (2015) 21:389–401.e1. doi: 10.1016/j.bbmt.2014.12.001 25529383 PMC4329079

[10] Qiu Y Hong J Peng R. Manifestation of clinical categories of ocular graft-versus-host disease. J Ophthalmol. (2018) 2018:6430953. doi: 10.1155/2018/6430953 30159166 PMC6109493

[11] Dietrich-Ntoukas T Cursiefen C Westekemper H Eberwein P Reinhard T Bertz H. Diagnosis and treatment of ocular chronic graft-versus-host disease: report from the german-Austrian-swiss consensus conference on clinical practice in chronic GVHD. Cornea. (2012) 31:299–310. doi: 10.1097/ICO.0b013e318226bf97 22157574

[12] Ogawa Y Kim SK Dana R Clayton J Jain S Rosenblatt MI. International chronic ocular graft-vs-host-disease (GVHD) consensus group: proposed diagnostic criteria for chronic GVHD (Part I). Sci Rep. (2013) 3:3419. doi: 10.1038/srep03419 24305504 PMC3851919

[13] Pathak M Diep PP Lai X Brinch L Ruud E Drolsum L. Ocular Findings and Ocular Graft-versus-Host Disease after Allogeneic Stem Cell Transplantation without Total Body Irradiation. Bone Marrow Transplant. (2018) 53:863–72. doi: 10.1038/s41409-018-0090-z PMC6039390 29382955

[14] Kantor NB Tovar A Wang T Galor A. How does ocular graft-versus-host disease fit under the dry eye umbrella? A review. Clin Experiment Ophthalmol. (2024) 52:167–85. doi: 10.1111/ceo.14347 PMC10939887 38204146

[15] Ramos-Casals M Brito-Zerón P Bombardieri S Bootsma H De Vita S Dörner T. EULAR recommendations for the management of sjögren’s syndrome with topical and systemic therapies. Ann Rheumatol Dis. (2020) 79:3–18. doi: 10.1136/annrheumdis-2019-216114 31672775

[16] Jonsson R Brokstad KA Jonsson MV Delaleu N Skarstein K. Current concepts on sjögren’s syndrome - classification criteria and biomarkers. Eur J Oral Sci. (2018) 126 Suppl 1:37–48. doi: 10.1111/eos.12536 30178554 PMC6586012

[17] Jonsson R Vogelsang P Volchenkov R Espinosa A Wahren-Herlenius M Appel S. The complexity of sjögren’s syndrome: novel aspects on pathogenesis. Immunol Lett. (2011) 141:1–9. doi: 10.1016/j.imlet.2011.06.007 21777618

[18] Pérez RL Pérez-Simón JA Caballero-Velazquez T Flores T Carrancio S Herrero C. Limbus damage in ocular graft-versus-host disease. Biol Blood Marrow Transplant. (2011) 17:270–3. doi: 10.1016/j.bbmt.2010.08.008 20800691

[19] Herretes S Ross DB Duffort S Barreras H Yaohong T Saeed AM. Recruitment of donor T cells to the eyes during ocular GVHD in recipients of MHC-matched allogeneic hematopoietic stem cell transplants. Invest Ophthalmol Vis Sci. (2015) 56:2348–57. doi: 10.1167/iovs.14-15630 PMC4406104 25655798

[20] He J Zheng F Zhang L Cai J Ogawa Y Tsubota K. Single-cell RNA-sequencing reveals the transcriptional landscape of lacrimal gland in GVHD mouse model. Ocul Surf. (2024) 33:50–63. doi: 10.1016/j.jtos.2024.04.006 38703817

[21] Baldini C Luciano N Tarantini G Pascale R Sernissi F Mosca M. Salivary gland ultrasonography: A highly specific tool for the early diagnosis of primary sjögren’s syndrome. Arthritis Res Ther. (2015) 17:146. doi: 10.1186/s13075-015-0657-7 26022533 PMC4461980

[22] Yang T Delli K Coumou AD van der Vegt B Kroese FGM Bootsma H. The lacrimal gland in sjögren’s syndrome: can we unravel its mystery using ultrasound? Clin Exp Rheumatol. (2022) 40:2428–33. doi: 10.55563/clinexprheumatol/z1lzb1 36377565

[23] Giovagnorio F Pace F Giorgi A. Sonography of lacrimal glands in sjögren syndrome. J Ultrasound Med Off J Am Inst Ultrasound Med. (2000) 19:505–9. doi: 10.7863/jum.2000.19.8.505 10944035

[24] De Lucia O Zandonella Callegher S De Souza MV Battafarano N Del Papa N Gerosa M. Ultrasound assessment of lacrimal glands: A cross-sectional study in healthy subjects and a preliminary study in primary sjögren’s syndrome patients. Clin Exp Rheumatol. (2020) 38 Suppl 126:203–9. 33095143

[25] Seceleanu A Pop S Preda D Szabo I Rogojan L Seceleanu R. Ultrasound features of lacrimal gland in sjogren’s syndrome: case report. Acta Clin Croat. (2012) 51 Suppl 1:135–40. 23431740

[26] Karadeniz H Cerit M Güler AA Salman RB Satış H Yıldırım D. Lacrimal gland ultrasonography and elastography as a diagnostic and activity tool for primary sjögren’s syndrome. Int J Rheumatol Dis. (2023) 26:1083–90. doi: 10.1111/1756-185X.14702 37137730

[27] Amescua G Ahmad S Cheung AY Choi DS Jhanji V Lin A. Dry eye syndrome preferred practice pattern^®^ . Ophthalmology. (2024) 131:P1–P49. doi: 10.1016/j.ophtha.2023.12.041 38349301

[28] Whitcher JP Shiboski CH Shiboski SC Heidenreich AM Kitagawa K Zhang S. Simplified quantitative method for assessing keratoconjunctivitis sicca from the sjögren’s syndrome international registry. Am J Ophthalmol. (2010) 149:405–15. doi: 10.1016/j.ajo.2009.09.013 PMC3459675 20035924

[29] Nichols JJ Berntsen DA Mitchell GL Nichols KK. An assessment of grading scales for meibography images. Cornea. (2005) 24:382. doi: 10.1097/01.ico.0000148291.38076.59 15829792

[30] Yonetsu K Takagi Y Sumi M Nakamura T Eguchi K. Sonography as a replacement for sialography for the diagnosis of salivary glands affected by sjögren’s syndrome. Ann Rheumatol Dis. (2002) 61:276–7. doi: 10.1136/ard.61.3.276 PMC1754034 11830440

[31] Milosevic S Bachnick B Karim K Bornkamm GW Witter K Gerbitz A. Identification of MHC II-restricted minor histocompatibility antigens after HLA-identical stem-cell transplantation. Transplantation. (2010) 90:1030. doi: 10.1097/TP.0b013e3181f5470c 20802400

[32] Ogawa Y Okamoto S Wakui M Watanabe R Yamada M Yoshino M. Dry eye after haematopoietic stem cell transplantation. Br J Ophthalmol. (1999) 83:1125–30. doi: 10.1136/bjo.83.10.1125 PMC1722843 10502571

[33] Ogawa Y Kuwana M. Dry eye as a major complication associated with chronic graft-versus-host disease after hematopoietic stem cell transplantation. Cornea. (2003) 22:S19–27. doi: 10.1097/00003226-200310001-00004 14703704

[34] Uchino M Ogawa Y Uchino Y Mori T Okamoto S Tsubota K. Comparison of stem cell sources in the severity of dry eye after allogeneic haematopoietic stem cell transplantation. Br J Ophthalmol. (2012) 96:34–7. doi: 10.1136/bjophthalmol-2011-300514 22053104

[35] Kamoi M Ogawa Y Uchino M Tatematsu Y Mori T Okamoto S. Donor–recipient gender difference affects severity of dry eye after hematopoietic stem cell transplantation. Eye. (2011) 25:860–5. doi: 10.1038/eye.2011.73 PMC3178168 21475315

[36] Worel N. ABO-mismatched allogeneic hematopoietic stem cell transplantation. Transfus Med Hemother. (2016) 43:3–12. doi: 10.1159/000441507 27022317 PMC4797460

[37] Ocular surface system alterations in ocular graft-versus-host disease: all the pieces of the complex puzzle | SpringerLink (Accessed 2023-08-20). 10.1007/s00417-019-04301-6 30944986

[38] Giannaccare G Bonifazi F Sessa M Dan E Arpinati M Fresina M. Ocular Surface Analysis in Hematological Patients before and after Allogeneic Hematopoietic Stem Cell Transplantation: Implication for Daily Clinical Practice. Eye. (2017) 31:1417–26. doi: 10.1038/eye.2017.78 PMC5639194 28524885

[39] Giannaccare G Bonifazi F Sessa M Fresina M Arpinati M Bandini G. Dry eye disease is already present in hematological patients before hematopoietic stem cell transplantation. Cornea. (2016) 35:638–43. doi: 10.1097/ICO.0000000000000747 26807906

[40] Cheng K-C Tu H-P Lin T-H Cheng K-H. The incident ocular diseases related to chemotherapy in cancer patients are associated with increasing risk of incident stroke. Acta Cardiol Sin. (2023) 39:435–48. doi: 10.6515/ACS.202305_39(3).20221005A PMC10203719 37229341

[41] al-Tweigeri T Nabholtz JM Mackey JR. Ocular toxicity and cancer chemotherapy. A review. Cancer. (1996) 78:1359–73. doi: 10.1002/(SICI)1097-0142(19961001)78:7<1359::AID-CNCR1>3.0.CO;2-G 8839540

[42] Jack MK Hicks JD. Ocular complications in high-dose chemoradiotherapy and marrow transplantation. Ann Ophthalmol. (1981) 13:709–11. 7020552

[43] Yamane M Ogawa Y Mukai S Yaguchi S Kamijuku H Inaba T. Functional role of lacrimal gland fibroblasts in a mouse model of chronic graft-versus-host disease. Cornea. (2018) 37:102–8. doi: 10.1097/ICO.0000000000001411 29053559

[44] Ogawa Y Shimmura S Dogru M Tsubota K. Immune processes and pathogenic fibrosis in ocular chronic graft-versus-host disease and clinical manifestations after allogeneic hematopoietic stem cell transplantation. Cornea. (2010) 29 Suppl 1:S68–77. doi: 10.1097/ICO.0b013e3181ea9a6b 20935546

