# Supplementary material for: Application of lacrimal gland ultrasonography in the evaluation of chronic ocular graft-versus-host-disease
Source: Front Immunol. 2025 Feb 5;16:1490390. doi: 10.3389/fimmu.2025.1490390 (PMC11836017; doi:10.3389/fimmu.2025.1490390)
Supplement: Supplementary file 1 [file Table1.docx]

**Supplemental information**

Classified by NIH CC 2014 criteria

All patients were assigned to two different groups (oGvHD and non-oGvHD). The demographics and transplantation characteristics of the patients are given in **Table S1**. Similar to the results with ICCGVHD criteria, except that the duration after allo-HSCT were remarkably longer in oGvHD patients than non-oGvHD patients (median with interquartile deviation: 416(227,714) vs 211(169,397), H=5.063, p＜0.05), there were no significant difference in other demographics and transplantation prameters including age, gender, donor-recipient gender combination, HLA matching, and ABO matching.

**TABLE | S1** Demographics and transplantation characteristics of patients after allo-HSCT classified by NIH CC 2014 criteria

| Variables | oGvHD(n=40) | Non-oGvHD(n=17) | H/F/χ² | P-value |
| --- | --- | --- | --- | --- |
| Age (years) | 40.35±11.497 | 39.24±12.726 | F=0.351 | 0.556 |
| Gender |  |  | χ²=0.192 | 0.661 |
| Female | 19(47.5%) | 7(41.2%) |  |  |
| Male | 21(52.5%) | 10(58.8%) |  |  |
| Duration after allo-HSCT | 416(227,714) | 211(169,397) | H=5.063 | **0.024^*^** |
| Donor-recipient HLA matching, n (%) |  |  | χ²=1.845 | 0.174 |
| Related HLA-identical donor | 17(42.5%) | 4(23.5%) |  |  |
| Haplp-identical family donor | 23(57.5%) | 13(76.5%) |  |  |
| Donor-recipient ABO matching, n (%) |  |  | χ²=0.914 | 0.822 |
| ABO-compatible | 18(15%) | 9(52.9%) |  |  |
| Major ABO-incompatible | 13(32.5%) | 6(35.3%) |  |  |
| Minor ABO-incompatible | 4(10.0%) | 1(5.9%) |  |  |
| Major&minor ABO-incompatible | 5(12.5%) | 1(5.9%) |  |  |
| Donor-recipient gender combination, n (%) |  |  | χ²=5.571 | 0.134 |
| Male to male | 4(10%) | 6(35.3%) |  |  |
| Female to female | 5(12.5%) | 2(11.8%) |  |  |
| Male to female | 14(35.0%) | 4(23.5%) |  |  |
| Female to male | 15(37.5%) | 4(23.5%) |  |  |
| Missing | 2(5.0%) |  |  |  |

Compared to non-oGvHD patients, oGvHD patients showed lower average of sight (H=4.849, p＜0.05), Schirmer test (H=32.860, p＜0.01), TBUT (H=5.752, p＜0.05), tear meniscus height (H=11.700, p＜0.01) and higher average of OSDI (F=9.575, p＜0.01), corneal staining scores (χ²=15.765, p＜0.05), nasal conjunctival staining scores (χ²=9.539, p＜0.05), temporal conjunctival staining scores (χ²=9.285, p＜0.05) and staining total scores (χ²=20.472, p＜0.05) (**Table S2**). There was no difference between groups in meibomian gland gland dropout and its grade, either.

**TABLE | S2** Ophthalmic parameters of patients after allo-HSCT classified by NIH CC 2014 criteria

| Variables | oGvHD(n=40) | Non-oGvHD (n=17) | H/F/χ² | P-value |
| --- | --- | --- | --- | --- |
| Sight | 0.60(0.50,1.00) | 1.00(0.60,1.00) | H=4.849 | **0.028^*^** |
| intra-ocular tension | 15.05(13.25,19.00) | 14.25(12.00,19.00) | H=0.487 | 0.185 |
| OSDI | 37.46±23.43 | 61.00±13.50 | F=9.575 | **0.003^*^** |
| Schirmer test | 2.50(1.25,5.75) | 15.50(14.00,18.50) | H=32.860 | **＜0.001^**^** |
| TBUT | 2.00(1.00,4.00) | 4.00(3.00,6.00) | H=5.752 | **0.016^*^** |
| Tear meniscus height | 0.16(0.11,0.18) | 0.22(0.15,0.30) | H=11.700 | **0.001^**^** |
| Corneal staining scores（≤6） |  |  | χ²=15.765 | **0.015^*^** |
| 0 | 14(35%) | 15(88.2%) |  |  |
| 1~3 | 11(27.5%) | 1(5.9%) |  |  |
| ≥4 | 15(37.5%) | 1(5.9%) |  |  |
| Nasal conjunctival staining scores（≤3） |  |  | χ²=9.539 | **0.023^*^** |
| 0 | 6(15.0%) | 9(52.9%) |  |  |
| 1~2 | 13(32.5%) | 2(11.8%) |  |  |
| 3 | 21(52.5%) | 6(35.3%) |  |  |
| Temporal conjunctival staining scores（≤3） |  |  | χ²=9.285 | **0.026^*^** |
| 0 | 12(30.0%) | 11(64.7%) |  |  |
| 1~2 | 10(25.0%) | 5(29.4%) |  |  |
| 3 | 18(45.0%) | 1(5.9%) |  |  |
| staining total scores（≤12） |  |  | χ²=20.472 | **0.039^*^** |
| 0 | 3(7.5%) | 6(35.3%) |  |  |
| 1~4 | 11(27.5%) | 9(52.9%) |  |  |
| 5~9 | 18(45.0%) | 1(5.9%) |  |  |
| ≥10 | 5(12.5%) | 0(0%) |  |  |
| Meibomian gland dropout (%) | 41.60(29.18,64.23) | 34.80(20.63,64.05) | H=0.989 | 0.320 |
| Grade of meibomian gland dropout（≤4） |  |  | χ²=5.102 | 0.277 |
| 0 | 0(0.0%) | 1(5.9%) |  |  |
| 1~2 | 28(70.0%) | 11(64.7%) |  |  |
| 3~4 | 10(25.0%) | 5(29.4%) |  |  |
| Missing | 2(5.0%) |  |  |  |

In lacrimal ultrasonography, the long diameter in oGvHD patients was shorter than that in non-oGvHD patients and showed significant difference (F=12.189, p＜0.01, **Table S3**). However, other parameters in lacrimal glands including thick diameter, homogeneity and parenchymal vascularization did not differ between the three groups, either.

**TABLE | S3** Lacrimal B-mode and Doppler ultrasonography evaluations of patients after allo-HSCT classified by NIH CC 2014 criteria

| Variables | oGvHD(n=40) | Non-oGvHD(n=17) | H/F/χ² | P-value |
| --- | --- | --- | --- | --- |
| Long diameter | 9.097±1.340 | 9.747±2.429 | F=12.189 | **0.001^**^** |
| Thick diameter | 3.444±0.818 | 3.806±1.118 | F=1.617 | 0.209 |
| Homogeneity |  |  | χ²=2.845 | 0.092 |
| Homogenous | 3(7.5%) | 4(23.5%) |  |  |
| Heterogeneous | 37(9.25%) | 13(76.5%) |  |  |
| Parenchymal vascularization |  |  | χ²=0.262 | 0.609 |
| Normal | 35(87.5%) | 14(82.4%) |  |  |
| Increased/decreased | 5(12.5%) | 3(17.6%) |  |  |

All the systemic immunosuppressive and immunomodulatory drugs the patients enrolled in our study took are in TABLE S4. In total, 78.9% (45/57) of patients were under systemic immunosuppresive therapy. 43.8% (25/57)of patients were under monotherapy, 21.05%(12/57) of patients were under two systemic immunosuppressive drugs and 14.04% (8/57) received immunosuppressive triple therapy.

**TABLE | S4** Systemic immunomodulatory and immunosuppressive therapy after alloHSCT: Different therapeutic regimes and number/percentage of patients.

| Systemic immunosuppressive therapy 45/57 |  | 78.9% |
| --- | --- | --- |
| Single-treatment regime 25/57 |  | 43.86% |
|  | -Cyclosporine A 2/57 | 3.51% |
|  | -Ruxolitinib 3/57 | 5.26% |
|  | -Prednisolone 7/57 | 12.28% |
|  | -Tacrolimus 1/57 | 1.75% |
|  | -Interferon 1/57 | 1.75% |
| Dual therapy 12/57 |  | 21.05% |
|  | -Ciclosporin A, Prednisolon 11/57 | 19.30% |
|  | -Mycophenolate mofetil, Prednisolon 3/57 | 5.26% |
|  | -Ruxolitinib, Prednisolon 3/57 | 5.26% |
|  | -Tacrolimus, Prednisolon 4/57 | 7.02% |
|  | -Others 2/57 | 3.51% |
| Triple therapy 8/57 |  | 14.04% |

**Ethics declarations**

Competing interests

The authors declare no conflict of interest.
